# Supplementary material for: The interactome of intact mitochondria by cross-linking mass spectrometry provides evidence for coexisting respiratory supercomplexes
Source: Mol Cell Proteomics. 2017 Dec 8;17(2):216–32. doi: 10.1074/mcp.RA117.000470 (PMC5795388; doi:10.1074/mcp.RA117.000470)
Supplement: Supplemental Data [file supp_RA117.000470_133922_0_supp_23978_fzffwf.zip › spectra_annotation/mito_spectra_annotation/267-1-6-1-11-1.pdf]

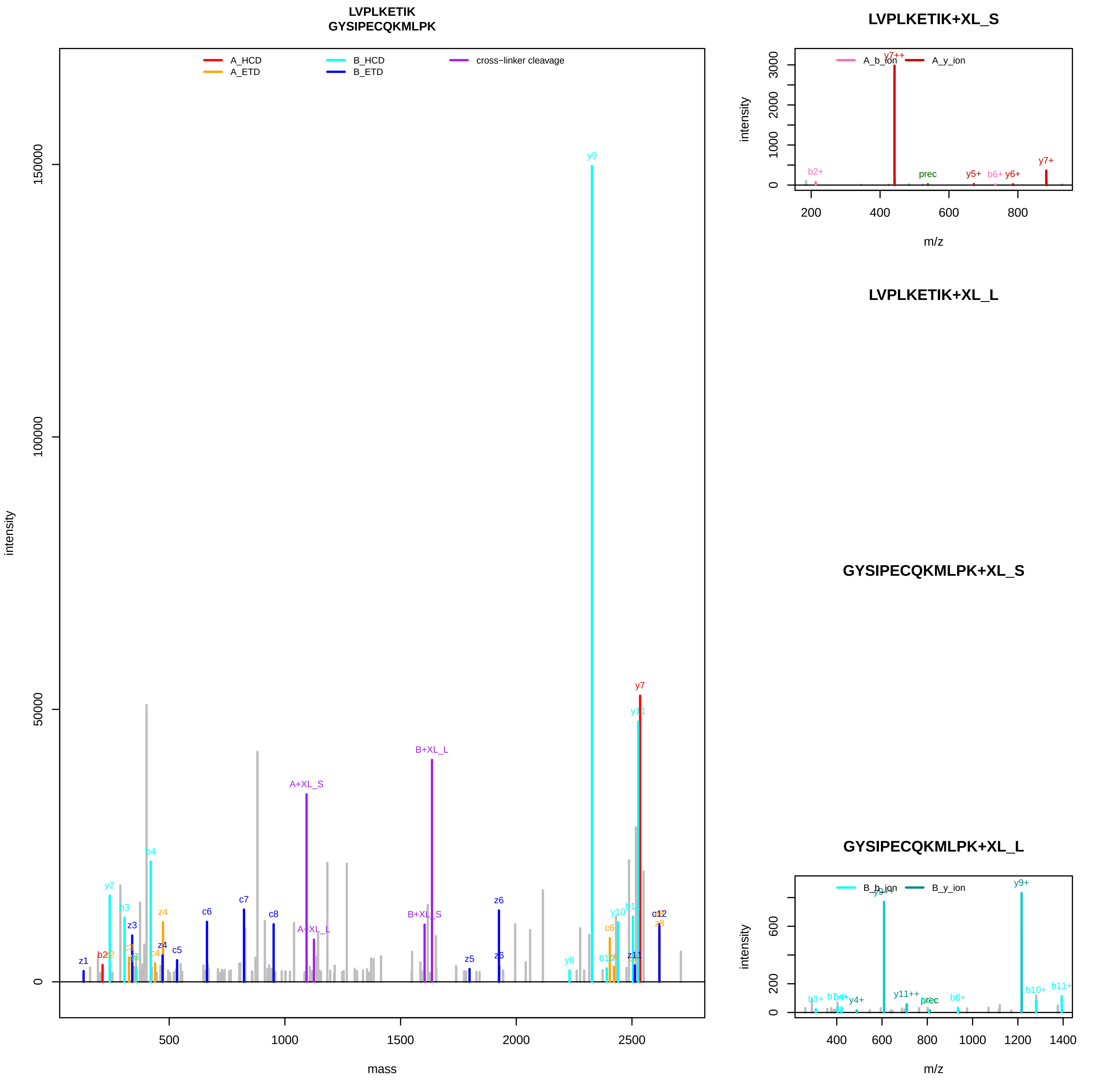
LVPLKETIK
GYSIPECQKMLPK
A_HCD
A_ETD
B_HCD
B_ETD
cross−linker cleavage
intensity
mass
y9
y7
y11
B+XL_L
A+XL_S
B+XL_S
A+XL_L
y2
b3
b4
z3
z4
c3
c4
c5
c6
c7
c8
z1
b2
z5
z6
y8
y10
c12
z8
z11
LVPLKETIK+XL_S
A_b_ion
A_y_ion
y7++
b2+
prec
y5+
b6+
y6+
y7+
m/z
LVPLKETIK+XL_L
GYSIPECQKMLPK+XL_S
GYSIPECQKMLPK+XL_L
B_b_ion
B_y_ion
y9++
y9+
b3+
y4+
y11++
b8+
b10+
b11+
